# Ultra-Lightweight Fast Anomaly Detectors for Industrial Applications

**DOI:** 10.3390/s24010161

**Published:** 2023-12-27

**Authors:** Michał Kocon, Marcin Malesa, Jerzy Rapcewicz

**Affiliations:** 1 KSM Vision sp. z o.o., 01-142 Warsaw, Poland; 2 Institute of Automatic Control and Robotics, Warsaw University of Technology, 02-525 Warsaw, Poland

**Keywords:** anomaly detection, quality control, X-ray image processing

## Abstract

Quality inspection in the pharmaceutical and food industry is crucial to ensure that products are safe for the customers. Among the properties that are controlled in the production process are chemical composition, the content of the active substances, and visual appearance. Although the latter may not influence the product’s properties, it lowers customers’ confidence in drugs or food and affects brand perception. The visual appearance of the consumer goods is typically inspected during the packaging process using machine vision quality inspection systems. In line with the current trends, the processing of the images is often supported with deep neural networks, which increases the accuracy of detection and classification of faults. Solutions based on AI are best suited to production lines with a limited number of formats or highly repeatable production. In the case where formats differ significantly from each other and are often being changed, a quality inspection system has to enable fast training. In this paper, we present a fast method for image anomaly detection that is used in high-speed production lines. The proposed method meets these requirements: It is easy and fast to train, even on devices with limited computing power. The inference time for each production sample is sufficient for real-time scenarios. Additionally, the ultra-lightweight algorithm can be easily adapted to different products and different market segments. In this work, we present the results of our algorithm on three different real production data gathered from food and pharmaceutical industries.

## 1. Introduction

Pharmaceutical and food manufacturers must comply with restrictive norms to ensure the highest quality standards. These standards apply to both production and packaging. The delivery of an incorrectly sealed product or a product in damaged packaging can result in complaints and sometimes the withdrawal of the entire product series from the market. Consequently, this means financial losses for the manufacturer.

Machine vision technologies are increasingly taking over traditional manual or visual inspection methods, becoming the standard in the pharmaceutical and food manufacturing industries. These machine vision solutions eliminate the need for direct contact with the inspected product, guaranteeing consistent and repeatable results. Moreover, they are specifically designed to function efficiently in high-speed production lines. The incorporation of algorithms rooted in deep neural networks further expands the scope of automatic quality control applications.

The problem with using standard, widespread solutions arises in contract manufacturing when different formats are produced on a single production line (or in a single production run). Defect detection models need to be adjusted (retrained) for each new format, which, when formats change two or three times a day, can result in long production downtime and system start-up errors. An example of the mentioned production type is packaging pharmaceuticals in blisters or bottle capping in the bottling industry. The diversity of tablets, capsules, bottles, and caps makes the fault detection algorithm cumbersome and time-consuming. This creates the need to use fast-to-operate and quick-to-set models for defect detection.

To address all of the objectives mentioned above, a fast, universal, adaptive, and ultra-lightweight anomaly detector was created. The effectiveness and adaptability of the proposed solution are demonstrated in the example of quality control of drugs in blisters, quality control of liquid packaging closures, and quality control of foreign bodies in food packaging using X-ray imaging.

This paper is organized as follows: Following the introduction is the section discussing related works. In Section 3, the design of our fast and lightweight system is presented with a detailed description of its parts: feature extractor, feature preprocessing, anomaly detector, and training procedure. Section 4 contains our experimental results compared with other anomaly detection models. Then, a use case with X-ray images is presented. This article concludes with a summary and future work descriptions.

## 2. Related Works

### 2.1. Importance of Quality Control in Industry

Quality control plays a significant role in the industry, being a key element in ensuring that products are manufactured to specified standards and meet customer expectations. It is a process that involves the systematic monitoring, measurement, and evaluation of all stages of production to detect any nonconformities or defects. There are several critical reasons for the need for quality control. Firstly, it guarantees that the products are safe for users and do not threaten health or life. Secondly, it ensures the consistency and repeatability of products, which translates into their quality and durability. Finally, quality control helps reduce costs by minimizing defects and waste of raw materials, increasing the company’s competitiveness in the market. In today’s global business environment, quality control is an essential part of any industry that strives for success and customer satisfaction [1,2].

Production quality control is based on various methods and techniques that aim to ensure the quality of products and eliminate possible defects and nonconformities. Visual inspection is a basic form of quality control that involves the visual examination of products to detect surface defects, aesthetic defects, or other apparent problems. It can be performed by a human but also with computer vision.

### 2.2. Computer Vision Quality Control

Computer vision quality control uses cameras and software to monitor and evaluate product quality automatically. It has many advantages that make it an effective quality control tool. These include automation (inspection takes place without human intervention), speed, accuracy, repeatability (the machine does not lose concentration like a human does), the possibility of inspecting many parameters (one photo can detect many types of defects), cost reduction (vision systems are flexible), and traceability and documentation (it is easy to document defects, which can make it easier to look for their causes). Examples of applications of machine vision in industrial quality control can be found in [3,4,5,6,7].

### 2.3. Artificial Intelligence Tools in Vision Systems

There are numerous artificial intelligence methods used in vision systems in many different fields, e.g., Viola–Jones cascade method [8], SIFT or SURF feature descriptors [9], color and texture histograms [10], support vector machines (SVM) [11], decision tree algorithms [12], and finally, neural networks [13,14,15]. Neural networks are commonly used in vision systems for several vital reasons [16,17]:

- Ability to learn: Neural networks, especially deep neural networks, can learn from data. As a result, they can adapt to various patterns in images and perform complex visual analysis tasks with proper training.
- Detecting caesuras: Neural networks can detect abstract and complex caesuras in visual data, such as objects, textures, shapes, and patterns. This makes them ideal for object recognition, image classification, and defect detection tasks.
- Scalability: Neural networks can be adapted to different tasks and levels of complexity. They can be used for simple tasks, such as recognizing characters in photos, and for more advanced ones, such as autonomous vehicles moving in a natural environment.
- Efficiency: Over the last few years, there have been significant developments in neural networks, making them increasingly efficient and precise. Modern neural network architectures, such as convolutional neural networks (CNNs), are particularly effective in image analysis.
- Understanding the context: Neural networks can consider the context in the analysis of images. They can recognize objects in the context of other objects or elements of the environment, which is essential in more advanced applications, such as facial recognition or autonomous navigation.
- Adaptation to changing conditions: Neural networks can be trained on a wide range of data, which allows them to cope with different lighting conditions, perspectives, or noise. This makes them useful in real-world applications where conditions can vary.
- Availability of tools and frameworks: Many machine learning tools and frameworks make it easier to work with neural networks, making them accessible to a wide range of people and companies.

Thanks to these features, neural networks have become an extremely effective tool in image analysis and visual processing, used in many fields, such as medicine, industry, transport, and entertainment. For this reason, neural networks have found applications in industrial quality control as anomaly detectors.

### 2.4. Anomaly Detectors

Anomaly detectors are tools used to identify abnormal behavior, patterns, or data in a dataset [18,19,20]. They have advantages and disadvantages that can affect their effectiveness in different contexts. The most important advantages of these detectors are as follows [21,22]: 1. Detection of unknown anomalies: Anomaly detectors can detect anomalies that were not previously known or included in the models, which is especially useful in situations where new types of anomalies appear. 2. Minimal need for data labeling: Unlike many machine learning methods, anomaly detectors do not require large datasets with anomaly labels. Often, access to normal data is enough to train the model. 3. Application in various fields: anomaly detectors have wide applications in various fields, such as cybersecurity, medicine, finance, and industry, as well as in monitoring IoT systems and devices.

However, the disadvantages include the following:

- High rate of false positives: One of the main problems with anomaly detectors is the propensity to generate false positives (incorrectly detecting anomalies on nondefective samples) [23].
- Lack of interpretability: With more advanced anomaly detector models, such as deep neural networks, it is not easy to understand why a given model considers certain data to be anomalies. Lack of interpretability can be a problem in cases where decisions need to be understood by humans.
- Dependence on training data: The performance of anomaly detectors can be highly dependent on the quality and representativeness of training data. The model may not perform well if the training data does not reflect actual conditions or is not sufficiently varied.
- The need to update the model: As the situation changes, the anomaly detection models may need to be updated regularly to continue detecting new types of anomalies.
- Difficulty in choosing the correct hyperparameters: Choosing the right hyperparameters for anomaly detection models can be challenging and require experimentation.

### 2.5. Anomaly Detection Tools

There are numerous methods for detecting anomalies in the industrial application of quality control [24]. These are, for example, the following: 1. Statistical methods of anomaly detection: These methods are based on statistical data analysis and detect anomalies based on deviations from the norm. Examples include the Grubbs test, the Chauvenet test, and the deviation from normal distribution test [25]. 2. Distance-based methods: These techniques measure the distance between data and its centroids or other reference points and detect anomalies based on the large distance from common patterns. Examples are the k-nearest neighbors method and the k-medoid method [26]. 3. Model-based methods: These techniques create models that describe the typical behavior of the data and identify anomalies as observations that do not fit these models. Examples include Gaussian Mixture Models (GMMs) and Hidden Markov Models (HMMs) [27]. 4. Machine-learning-based methods: Machine learning methods such as Isolation Forests, One-Class SVMs, or autoencoders can be used to train models on data labeled as “normal” and detect anomalies as deviations from this normal pattern [28]. 5. Neural-network-based methods: Neural networks, in particular autoencoders and recursive neural networks (RNNs), can be used to detect anomalies by modeling and comparing input data with their reconstructions [29,30].

### 2.6. Autoencoders

One of the common methods for detecting anomalies in quality control is an autoencoder. Autoencoders are artificial neural networks used to detect and learn data representations. Their primary purpose is to compress the input into a lower-dimensional representation and then play it back to its original form [31].

The general idea of how autoencoders work is as follows [32,33]: 1. Encoder: The first part of the autoencoder is called the “encoder”. In this phase, the input data are processed to create a lower-dimensional representation, usually called a “code” or “hidden representation”. This code is typically much smaller than the input data and contains important data characteristics. 2. Decoder: The second part of the autoencoder is called the “decoder”. In this phase, the hidden representation is processed to recreate the input. The goal is to reproduce the original data as accurately as possible. 3. Cost function: Autoencoders are trained to minimize the difference between the input data and its reconstruction. The most commonly used cost function is the mean squared error (MSE), which measures the mean squared difference between pixel values (in the case of images) or other similarity measures in other domains. 4. Learning: In the learning process, the neural network adjusts the weights and parameters of its layers to minimize the cost function. This means the network tries to find an optimal hidden representation containing relevant information about the input data.

Once the autoencoder is trained, it can be used for a variety of tasks, such as reducing dimensions (by using code as a new data representation), removing noise from data (by giving noisy data as input and using the decoder), generating new data (by sampling from the code space), or anomaly detection (by comparing the input data with their reproduction). However, autoencoders have several important disadvantages [34,35]: (1) Uninterpretability: The hidden representation created by the autoencoder is usually difficult to interpret, making it difficult to understand what features are represented by the hidden neurons. (2) Long learning time: Autoencoders can take a long time to train, especially for large datasets and deep network architectures. (3) Overfitting: As with other machine learning models, autoencoders are susceptible to overfitting, especially in small datasets. They may learn to replicate training data too closely, leading to poor generalization. (4) Initialization dependence: The autoencoder training result may depend on the initial values of the network weights and parameters, which may make the training process more unstable. (5) Difficulty in choosing the exemplary architecture: Choosing the right autoencoder architecture (number of layers, number of neurons in hidden layers, etc.) can take time and require experimentation.

### 2.7. The Use of Autoencoders for Quality Control in the Industry

The following can be mentioned among the described cases of using autoencoders for quality control in industry. Nguyen et al. developed a system for detecting defects in steel. They built their own dataset. They managed to reduce false detections from 6.8% to 1.5% compared with non-deep-learning methods. However, their work is theoretical, as this algorithm has yet to be tested online in production [36]. Similarly, researchers in [37] dealt with the detection of defects on the steel surface, proposing a new approach to building an autoencoder. They achieved good results but should have mentioned the speed criteria to implement their system in production. Researchers from the Technical University of Sofia, Bulgaria, have successfully used an autoencoder to test print quality compared with traditional methods [38]. Other examples of the use of autoencoders relate to testing the quality of electrode coating in the automotive industry [39], testing the quality of sheet metal using data obtained directly from production [40], controlling the printed circuit board using data from production [41] and on artificial data [42]. In addition, there are numerous other examples of using autoencoders to test quality in production tasks [43,44,45].

### 2.8. Autoencoders and X-ray

Autoencoders are also used in searching for anomalies in X-ray images. We now present some examples of such applications in the industrial area.

The obvious and historically primary application of X-rays is medicine. There are many works here that use autoencoders to look for anomalies in X-ray images, e.g., [46,47,48].

In industrial circles, X-ray is used to detect material defects or unwanted elements in other objects, which allows the detection of anomalies in the entire volume, not just on the surface. Among the works that use autoencoders for these purposes, it is worth mentioning [49], in which the quality of metal details is examined. However, this work is based on artificial data. Researchers in [50] used autoencoders to study X-ray images in the die casting industry. They achieved an efficiency of 97% compared with an SVM-based method. Quality inspection in the automotive industry is described in [51]. In this case, an autoencoder for looking for anomalies in X-ray images was used to find air bubbles. The method achieved 91–94% efficiency.

### 2.9. Conclusions from the Literature Review

A review of the scientific literature indicates that there are practical solutions using autoencoders in anomaly detection tasks in the industry. High prediction accuracy is achieved, and the application in terms of industrial sectors is wide. However, typical applications do not involve fast processes that frequently change product formats and where models often need to be trained on new samples. None of the works found deal with the prediction speed of new samples, which is crucial in working on the production line. Researchers mention the neural network learning time parameter, which is also very important. However, it must be kept in mind that in high-volume quality control applications, defect detection times must be tens of milliseconds to ensure that the inspection process does not slow production.

There is a need for solutions that are both easy and swift to implement, particularly in scenarios such as installing a new machine or introducing a new product. The ideal solution should have a rapid startup, be quick to adapt during training (for instance, when transitioning a machine for a different product), and efficiently integrate into industrial production settings. To optimize these processes, a universal and preconfigured autoencoder (AE) model is essential. This model should be versatile enough to be applied across various products in diverse factories, with minimal installation and training times. Specifically, emphasis is placed on the model’s ability to handle small training data sizes, typical of autoencoders, and its ease of learning when introducing a new product on the production line. This simplicity is crucial for the line operator, who should be able to execute the learning process without delving into complex hyperparameters, eliminating the need for an in-depth understanding of learning concepts.

The quality control system we propose uses an autoencoder, which has been used both for visual control of the quality of tablets in the pharmaceutical industry, visual control of the quality of bottles in the bottling industry, or for detecting objects in food using X-rays.

The system is universal (the same model for different products, thus easy and cheap to implement), easy to train (does not require a large amount of data, labeling, or fake samples, and the machine operator can perform training), and fast in action (runs in real time on the machine).

## 3. The Methodology

### 3.1. System Overview

**Figure 1:**
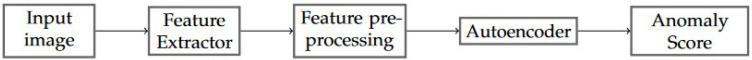
Our anomaly detection system’s main components.

Our anomaly detection system consists of three components: feature extractor, feature preprocessing block, and autoencoder. The architecture of our system is shown in Figure 1.

The feature extractor is responsible for capturing relevant information from the input image. We use a pretrained CNN to obtain well-generalized features. The next step is feature scaling, which standardizes the extracted features, ensuring they are on the same scale. Scaled features are passed to the autoencoder, which creates the reconstruction of input features. Finally, reconstruction loss is calculated as the difference between input and reconstruction features. The value of reconstruction loss is treated as an anomaly score. The training of our system is limited to setting the parameters of the feature scaler and training the autoencoder, which enables fast training on edge devices with limited computing power.

### 3.2. Features Extraction

The fundamental objective of the feature extractor is extracting pertinent information from the input image. Our system uses a CNN architecture MobileNetV2 [52] as a backbone. The network’s input shape is dynamically determined internally by our system, an adaptive process aligning the architecture with the original dimensions of the input images. The input dimensions of the neural network are determined from a predefined set of options. These options were prepared based on the most common image shapes existing in our computer vision systems. This adaptability is integral to accommodating diverse image sizes and resolutions encountered in real-world applications. We leverage a pretrained instantiation of MobileNetV2, initially trained on the ImageNet dataset [53] with the width multiplier parameter set to 1.

Input images of the feature extractor network have to be preprocessed so the features can be extracted properly. For MobileNetV2, the only required preprocessing is image scaling into the range [0.0, 1.0].

MobileNetV2’s primary objective is a classification task; to harness its feature extraction prowess, we discard the final layers of the model. Instead, we select the “block_15_add” layer within the network as the output layer.

### 3.3. Feature Preprocessing Block

In the context of our study, it is imperative to preprocess extracted features to align them with the proposed autoencoder architecture. Assuming an input shape of (224, 224, 3) for the CNN backbone, the features extracted from it are initially organized in a 7 × 7 × 160 tensor structure. This tensor retains spatial information, which, at this particular stage, is not necessary. Consequently, a flattening operation is performed, transforming the tensor into a vector with a dimensionality of 7840.

The next step is feature normalization, which ensures that all features have the same scale and will have a comparable impact on the autoencoder’s predictions. We use min–max normalization, rescaling each feature to the 0–1 range. Minimum and maximum values for each feature are dynamically computed during the training phase of the system. These values are then used during the inference phase to ensure that new sample’s features are comparable with the training ones.

### 3.4. Autoencoder

The proposed autoencoder architecture is a fully connected neural network with batch normalization layers and one dropout layer. The autoencoder was implemented in *TensorFlow* with the use of *Keras* layers. The final architecture of the autoencoder is a result of experiments on diverse datasets from different products and different market segments. This empirical approach ensures the adaptability and robustness of the final autoencoder design, substantiating its efficacy across a broad range of applications and domains. The structure of the autoencoder is presented in Table 1.

Reconstruction error is calculated as the difference between input features and the autoencoder output. The reconstruction error is calculated via the application of the mean squared error (MSE) metric. Following the reconstruction process, the reconstruction error undergoes polynomial transformation. The coefficients of the polynomial function are computed to align with a manually devised function designed to convert reconstruction error values into a representation that is more comprehensible to human interpretation. Subsequently, these transformed values are constrained within the interval [0, 0.99], resulting in the derivation of an anomaly score. In the majority of practical scenarios, it is observed that normal samples exhibit anomaly scores that tend to be lower than 0.25 after undergoing these sequential transformations.

The system’s input image is classified as abnormal if its anomaly score is larger than the threshold value. The threshold is set automatically based on the values of anomaly scores of training samples but can be fine-tuned manually by a human operator.

### 3.5. Training

The training of our system consists of two stages. In the first stage, all correct training images are preprocessed and then passed through the feature extractor. Minimum and maximum values for each feature are calculated and set as parameters for the min–max scaler, and based on these parameters, training features are scaled. The same configuration of the min–max scaler will be used on the new sample inference. Scaled training features are used to train the autoencoder network.

In the second stage, we train the autoencoder for 50 epochs. We use Adamax with a learning rate set to 0.001 as optimizer and MSE as loss function. the metric used for model evaluation is also MSE. Using the same metric for training and calculating reconstruction error ensures that the autoencoder will try to minimize anomaly scores on training samples.

In the realm of industrial applications, our system undergoes training, employing a dataset comprising no fewer than 200 images. The features extracted from these data enable the autoencoder to learn the intricate patterns and nuances present within the dataset of nonanomalous samples, thereby enhancing its capacity for discernment and classification in real-world industrial scenario.

## 4. The Experiments

### 4.1. Datasets

We evaluated our method on three datasets: one public dataset and two datasets gathered by vision systems installed on industrial production lines.

As a public dataset, we chose MVTec anomaly detection dataset [54,55], which focuses on industrial inspection. MVTec AD contains over 5000 images divided into 15 categories. Each category has its own training set with nonanomalous samples and a test set containing both anomalous and nonanomalous samples.

**Figure 2:**
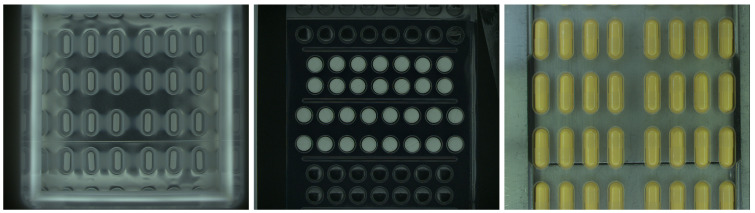
Images gathered by vision system installed on a machine for packaging pharmaceutical pills in blisters.

**Figure 3:**
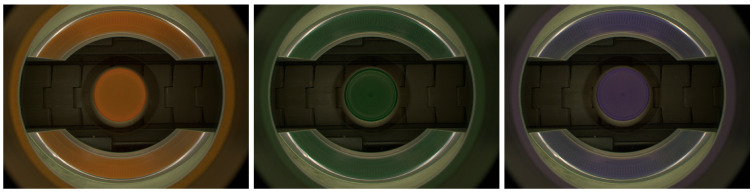
Images gathered by vision system installed on beverage production line. A 360-degree image of the bottle cap is obtained with a single camera with the use of mirrors.

**Figure 4:**
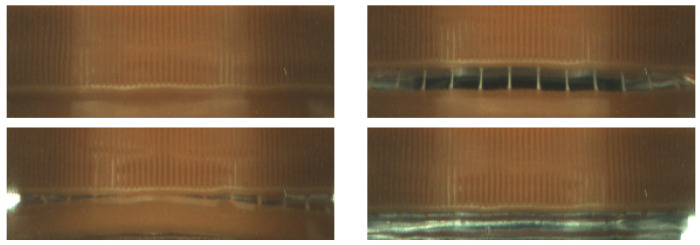
Sample normal (top left) and anomalous images from bottle cap dataset. Images are rectified (unwrapped) from Figure 3.

Two other datasets contain data collected on different production lines from different manufacturing domains: pharmaceutical and food markets. The first dataset contains images gathered by our vision system installed on machine for packaging pharmaceutical pills in blisters. The images of single pills are cropped from a full image based on predefined regions. Across the machines integrated with our optical quality control system, a diverse array of pills and capsules are packed into blisters, varying in terms of size, geometry, and coloration (Figure 2). Our anomaly detection system works for all pill–blister combinations. Images in the second dataset were acquired on the beverage production line. Our quality control system detects faults in bottle caps; a 360-degree image of the bottle cap is captured with a single camera. The caps, which are visible on mirrors, are remapped to rectangular images. See Figure 3 and Figure 4. The sizes and colors of controlled caps differ for different beverages, but all of them are controlled with our anomaly detection system (Figure 1). Both datasets preserve the same test and train set structure as each MVTec AD category.

The training set of the pills dataset contains 350 RGB images of white oblong pills on an aluminum background. All images have the shape (100, 210, 3). The test set contains 250 nonanomalous samples and 224 defective samples. Abnormal samples have different types of defects: scratches, cracks, improper pill orientation, missing part of the pill, or extra pill elements.

The bottle cap dataset consists of data from Inspect360+ visual inspection systems. Caps image are gathered with a single camera and transformed with the calibration procedure described in [56]. The training set contains 243 RGB images with the shape (280, 96, 3). The test set contains 160 normal and 132 abnormal images with defects: broken inviolability ring, absence of cap, and inclined cap.

**Figure 5:**
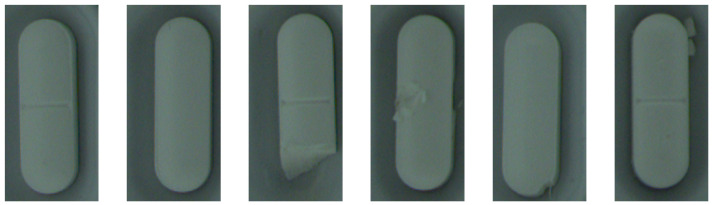
Sample normal (two most left) and anomalous images from pills dataset.

Samples from pills and bottle cap datasets are shown in Figure 4 and Figure 5.

### 4.2. Anomaly Detection Models

We compare our solution with FastFLow [57], EfficientAD [58], Deep Feature Kernel Density Estimation (DFKDE), DFM [59], PaDiM [60], and PathCore [24]. We used implementations provided by *anomalib* library [61] with default parameters. For FastFlow, we used a ResNet18 backbone, set the number of flow steps to 8, and only used 3 × 3 convolutional layers in flow steps. A small version of EfficentAD was used—EfficientAD-S with 384 channels on the output of the teacher network. For DFKDE, a ResNet18 was used as the backbone, and the number of PCA components was set to 16. For DFM, we used a ResNet50 backbone and set PCA to retain 97% of the original data variance. For PaDiM, we also used the ResNet18 backbone. We used the PatchCore-10% version of PatchCore algorithm with WideResNet50_2 used as the backbone. For all models, images were resized to (256, 256), and further cropping was omitted.

### 4.3. Metrics and Experimental Setup

For the evaluation of models, we selected two metrics used in anomaly detection tasks:

- AUROC;
- F1-score.

For the MVTec AD dataset, we report metrics as an average of metrics over all categories. We only evaluated our model on MVTec AD. For other models, we report values from anomalib documentation and original papers. Metrics achieved with anomalib implementations differ from those in the original papers. In comparison, we use both the original and anomalib results.

In industrial applications models, performance is not the only important metric; furthermore, in computer vision systems installed on production lines, other properties can be more critical. To compare different models’ applicability in the industrial environment, we report the models’ latency and time of training required to obtain high-accuracy results. We measure only the latency of models, not throughput, because in real-time systems, the analysis of samples is run one by one as they move on the conveyor belt. We run all experiments on the machine with only a CPU—Intel Core i7-10700K processor. We did not use GPU acceleration to mimic the production environment, where models are run on edge devices with limited computation power and no GPUs are available. When testing latency, we used OpenVino Toolkit [62] for all models to optimize the models’ inference time on the CPU.

### 4.4. Results

The results for anomaly detection performance are shown in Table 2. For FastFlow, EfficientAD, and PatchCore, we report two values for AUROC on the MVTec AD dataset, the first values originating from anomalib library documentation and the second ones from original papers. For EfficientAD and PatchCore, the results from anomalib implementation are close to the original results. For FastFlow, the difference is significant, which may be caused by the nonoptimal training strategy used to obtain the results, as stated in the anomalib documentation. For the other models in the configurations we used, there are no reported results on the MVTec AD dataset in official papers.

On the MVTec AD dataset, our model suppresses the DFKDE model and achieves results comparable to PaDiM and the anomalib implementation of FastFlow on both the AUROC and F1-score metrics. DFM, EfficientAd, and PatchCore achieve higher performance on selected metrics. AUROC, reported in the original FastFLow paper, also achieves higher scores than those obtained by our model. On our production datasets, all tested models achieve high or very high AUROC and F1-score. On the bottle cap dataset, most of the methods achieve perfect metrics, meaning no samples are misclassified.

Table 3 shows the latency and training time on our production datasets. Our method achieves the lowest latency and training times over all tested methods. Our method’s latency is 10–15× lower than the latency of methods with comparable anomaly detection performance and up to 125× lower than the PatchCore method, which has the highest mean accuracy over all tested datasets. The training time of our method is lower by an order or two orders of magnitude compared with other methods.

Our anomaly detection system achieves lower values for AUROC and F1-score metrics than state-of-the-art anomaly detection models. While alternative methodologies may achieve superior accuracy metrics, our approach yields results with sufficient precision, enabling deployment in highly repetitive manufacturing environments. Upon comparative analysis with existing techniques, our anomaly detection model distinguishes itself through its remarkable speed during both the training and inference phases. This heightened operational efficiency positions our model as a well-suited choice for integration within high-speed production lines, where swift decision making is imperative. However, the main drawback of the presented method is less accurate prediction compared with other methods.

### 4.5. X-ray: Use Case of Our Anomaly Detector

**Figure 6:**
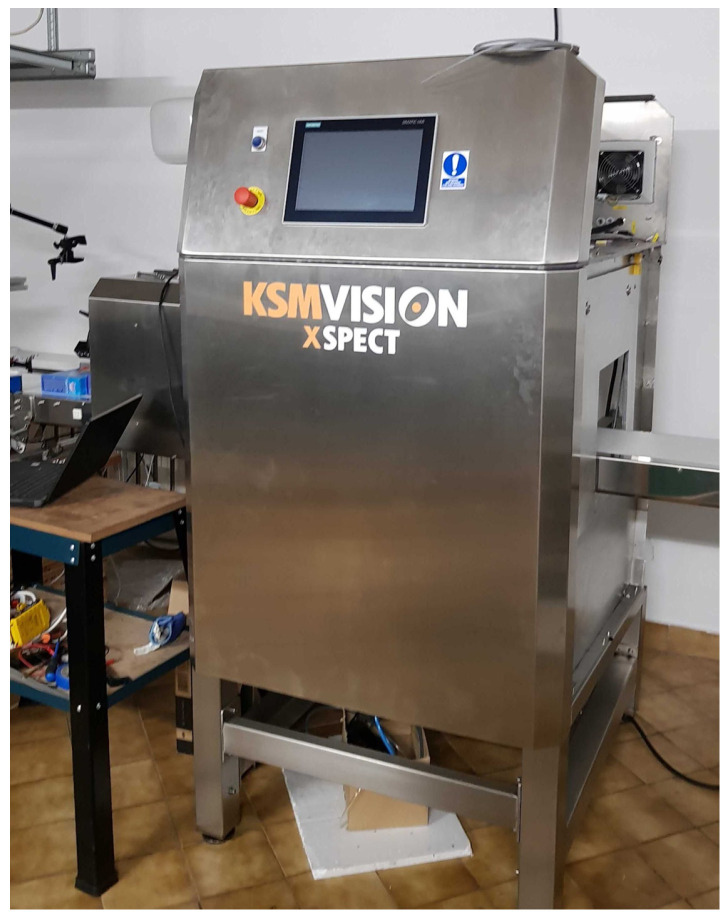
The KSM Vision Xspect device.

The developed anomaly detection system was used to search for foreign bodies in jars of pickled cucumbers. For this purpose, a device equipped with an X-ray source, a detector, a linear conveyor, and radiation protection covers was used (see Figure 6).

**Figure 7:**
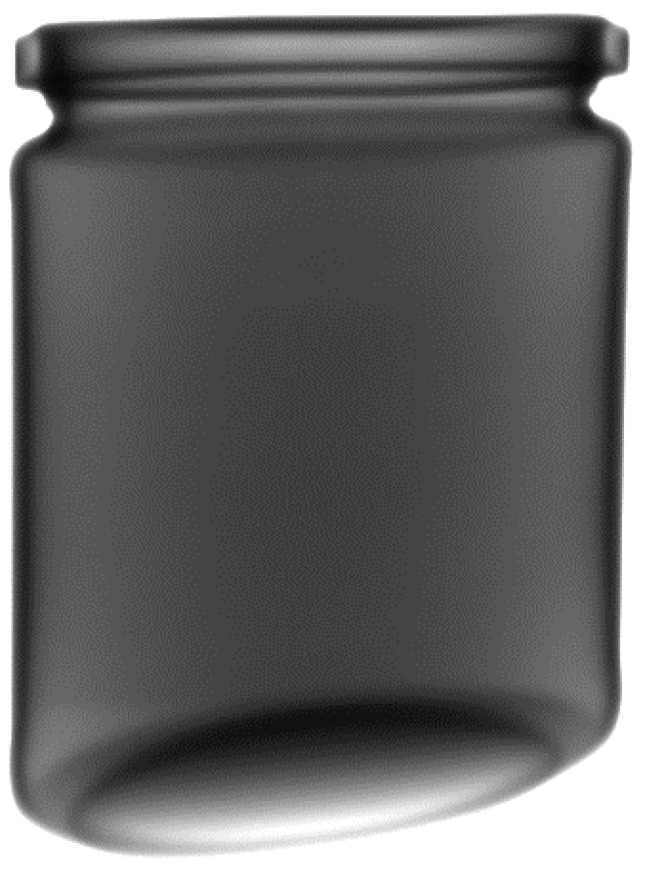
A normal jar (without any foreign body).

**Figure 8:**
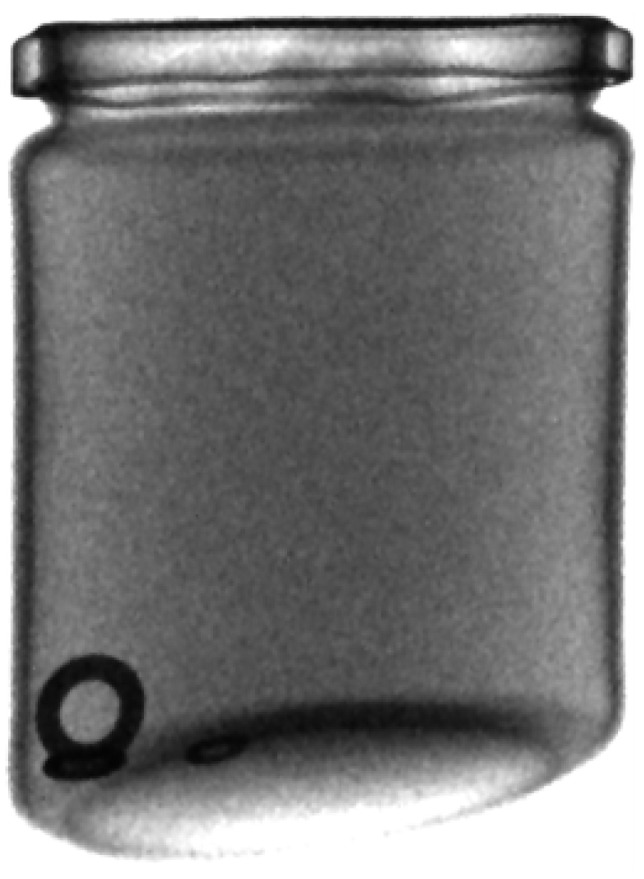
A defective jar (with several washers inside).

As part of the development of the foreign body detection system, several methods were used to compare them and select the best one. In each case, photos were taken of nondefective jars (Figure 7) and defective ones (Figure 8), i.e., those with various foreign bodies made of metal, rubber, and glass, and photos of these jars were taken. Some photos were used to train the models and the rest for testing. The size of the nondefective training set was 357 images, the defective testing set had 215, and the nondefective testing set had 200 images.

The same image preprocessing was used for all methods, i.e., jar localization by edge detection, limiting the data range by thresholding, gamma correction, and noise reduction.

The data prepared in this way were used for four methods: 1. Fine-tuning pretrained Xception, ResNet50, and InceptionV3 networks; 2. Developing own convolutional neural network; 3. Anomaly detection by background subtraction; 4. Using an autoencoder as an anomaly detector.

X-ray images are specific due to high noise rate and dynamic image depth range, so using fine-tuned pretrained models yielded results of only 68% in correct defect detecting. This is because these models were not trained on data resembling X-ray images. An artificial network designed by our own gave much better results (85% correct predictions), but in this case, the amount of training data was too small to achieve an even better result. A simple background subtraction algorithm was also used (the background was averaged over dozens of nondefective photos). This resulted in a high jar prediction efficiency of 95%. However, the high result of this method results from the use of features of a specific product, so it has little generalization, so it might not work on other products. Finally, the raw data were used to train the anomaly detector. The results in this case differ and depend on which part of the jar was examined. The middle part of the jar achieved 94% True Negative and 56% True Positive. The results are 90% True Positive, and 41% True Negative for the same part of the jar when the photos were divided into segments.

**Figure 9:**
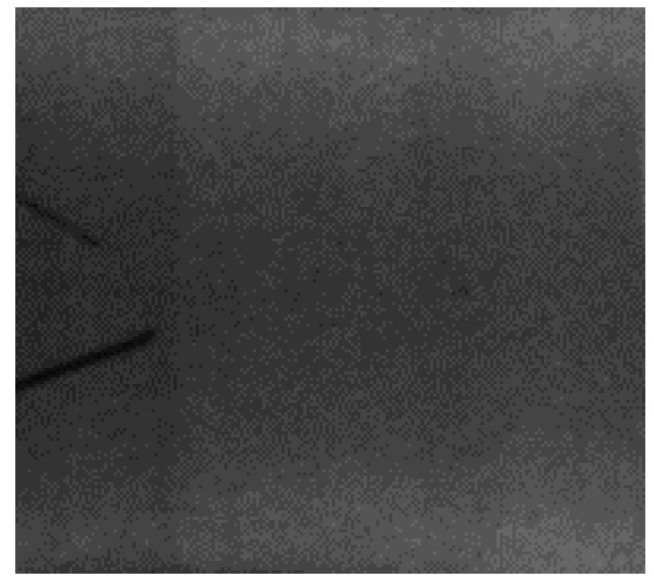
A cropped region of interest (ROI) with foreign body (a metal wire) seen on the left.

**Figure 10:**
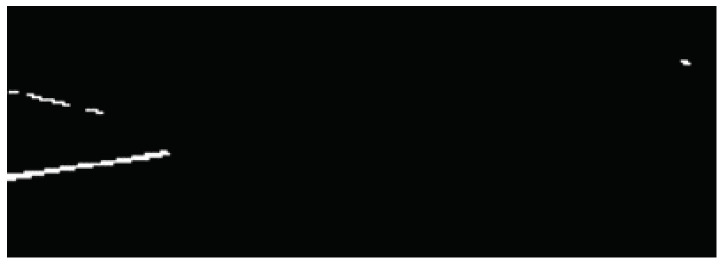
ROI from image Figure 9 preprocessed and resized.

Another experiment used more complex preprocessing of X-ray images containing the same jars (the same dataset as described above was exercised). Images were binarized using adaptive thresholding, with a threshold value to retain all foreign bodies. This also resulted in some minor background noise remaining in the binary image, but this prevented information about the foreign bodies from being lost (see Figure 9 and Figure 10).

Using this preprocessing, nondefect images (with jars containing no foreign bodies) were processed by the anomaly detector described in this paper. Firstly, images were used for feature detection by an extractor based on the OpenVino model (pretrained on the ImageNet dataset). These features were used to train an anomaly detector using an autoencoder. This algorithm was then tested on other sets of nondefective and defective jars. Although this method required a set of several faulty images to determine the threshold value, which is unusual for anomaly detectors, it still achieved good results. The minimum size of the training dataset for such an algorithm was also assessed. Table 4 shows the results of this comparison. For every set from 350 to 150 samples, the accuracy is greater than 90%. Only when the number of training samples is fewer than 150 does the algorithm classify all samples as good or bad. Applying an anomaly detector to a binary image made it possible to obtain an algorithm practically insensitive to very high noise, which is usually the case with X-ray images. The learning time achieved was 10 s for the set containing 357 samples.

For comparison, the results reported in [51] give an effectiveness of 91–94%. In this case, air bubbles are detected in the engine body. Researchers use a three-level autoencoder. The results are comparable, but the algorithm described requires a huge set of defective images for training, which may be challenging to meet in many applications. In [63], researchers trained the YOLOv4 model and achieved 94% efficiency when looking for foreign bodies in food in X-ray images. However, in the work in [64], which examined tire defects using X-ray, an autoencoder-based anomaly detector achieved an efficiency of at best 68%.

So far, the anomaly detector results require improvement, but some indicators are satisfactorily high. Work is still being carried out to improve the prediction efficiency of this anomaly detector in X-ray images. Since the anomaly detector only needs normal data, obtaining it is cheaper and more straightforward, which is essential in the case of a device such as an X-ray. Hence, there is a need to continue work and develop the anomaly detector model.

## 5. Conclusions and Future Works

In conclusion, this paper addresses the imperative need for stringent quality control in pharmaceutical and food manufacturing, encompassing both production and packaging processes. The utilization of machine vision technologies, coupled with deep neural network algorithms, has emerged as a pivotal standard, surpassing manual inspection methods. These technologies provide noncontact, consistent, and high-speed inspection capabilities.

However, challenges arise in contract manufacturing scenarios, where diverse formats necessitate frequent retraining of defect detection models. This recurrent format change, occurring multiple times a day, can lead to significant production downtime and system startup inefficiencies, especially in industries such as pharmaceutical blister packaging and bottle capping.

To surmount these challenges, we introduce an ultra-lightweight anomaly detector that is fast both in inference and training. This solution demonstrates its effectiveness in various quality control applications, including drugs in blisters, liquid packaging closures, and detecting foreign bodies in food packaging using X-ray imaging, as shown above.

In the future, we intend to train a feature extractor model from scratch on a large set of industrial data to increase the accuracy and ease of adaptation to production conditions. The current instantiation of the feature extractor employs training on the ImageNet dataset, facilitating the extraction of generalized features applicable to a broad spectrum of input images. However, the feature extraction can be enhanced by training the model on a dataset tailored to specific industrial domain. This will empower the model to generate features that are specifically attuned to the industrial data.

In summary, our proposed solution significantly advances the field, offering a versatile and efficient approach to quality control in diverse manufacturing settings. We anticipate that this work will pave the way for further innovations and enhancements in this critical domain.

## Figures and Tables

**Table 1:** Autoencoder architecture.

Layer Type	Neurons
Input	7840
Dropout	
Dense	128
BatchNormalization	
Dense	64
BatchNormalization	
Dense	64
BatchNormalization	
Dense	64
BatchNormalization	
Dense	256
BatchNormalization	
Dense	7840

**Table 2:** Anomaly detection performance. Metrics for MVTec AD dataset originate from anomalib documentation or official papers. When more than one value is reported, the first one originates from anomalib documentation and the second one from official papers. The best results are in bold.

	MVTec AD		Pills Dataset		Bottle Cap Dataset	
Method	AUROC	F1-Score	AUROC	F1-Score	AUROC	F1-Score
FastFlow	0.907/0.979	0.916	0.985	0.957	**1.0**	**1.0**
EfficientAD	**0.982**/0.988	0.970	0.961	0.932	**1.0**	**1.0**
DFKDE	0.762	0.872	0.954	0.905	0.992	0.969
DFM	0.936	0.943	0.990	0.975	**1.0**	**1.0**
PaDiM	0.891	0.916	0.993	0.956	0.996	0.996
PatchCore	0.980/**0.990**	**0.976**	**0.997**	**0.987**	**1.0**	**1.0**
Our	0.908	0.925	0.985	0.960	**1.0**	**1.0**

**Table 3:** The latency and training time on our production datasets achieved by different methods.

Method	Latency [ms]	Training Time—Pills Dataset [s]	Training Time—Bottle Cap Dataset [s]
FastFlow	50.3	3308	2072
EfficientAD	128.0	12,779	2091
DFKDE	24.7	123	117
DFM	47.6	165	143
PaDiM	35.6	138	127
PatchCore	438.4	7699	3741
Our	**3.5**	**13**	**10**

**Table 4:** Accuracy of the algorithm depending on the size of the training set size. Values are percentages, so they point out how many samples were classified correctly (“good” and “faulty” for nondefective and defective sets, respectively). In this case, the accuracy of the training set was below 100%. Values are approximately repeatable for sets greater than 100.

	Set Name or Number							
	Ref. Set	1	2	3	4	5	6	7
Training set size	357	306	257	208	154	106	57	10
Training set	97%	95%	95%	96%	97%	0%	0%	100%
Nondefective test set	96%	93%	94%	97%	98%	7%	0%	100%
Defective test set	88%	93%	92%	91%	91%	98%	100%	0%

## Data Availability

The source code and data used to support the findings of this study are available from the corresponding author upon request.

## References

[1] Montgomery D.C. (2005). Introduction to Statistical Quality Control.

[2] Avigdor Z., Kenett R.S. (2020). Quality 4.0—the challenging future of quality engineering. Qual. Eng..

[3] Villalba-Diez J., Schmidt D., Gevers R., Ordieres-Meré J., Buchwitz M., Wellbrock W. (2019). Deep learning for industrial computer vision quality control in the printing industry 4.0. Sensors.

[4] Nascimento R., Martins I., Dutra T.A., Moreira L. (2023). Computer vision based quality control for additive manufacturing parts. Int. J. Adv. Manuf. Technol..

[5] Longfei Z., Zhang L., Konz N. (2022). Computer vision techniques in manufacturing. IEEE Trans. Syst. Man Cybern. Syst..

[6] Jinjiang W., Fu P., Gao R.X. (2019). Machine vision intelligence for product defect inspection based on deep learning and Hough transform. J. Manuf. Syst..

[7] Luo Q., Fang X., Liu L., Yang C., Sun Y. (2020). Automated visual defect detection for flat steel surface: A survey. IEEE Trans. Instrum. Meas..

[8] Ciberlin J., Grbic R., Teslić N., Pilipović M. Object detection and object tracking in front of the vehicle using front view camera. Proceedings of the 2019 Zooming Innovation in Consumer Technologies Conference (ZINC).

[9] Monika B., Kumar M., Kumar M. (2021). 2D object recognition: A comparative analysis of SIFT, SURF and ORB feature descriptors. Multimed. Tools Appl..

[10] Zhou W., Gao S., Zhang L., Lou X. (2020). Histogram of oriented gradients feature extraction from raw bayer pattern images. IEEE Trans. Circuits Syst. II Express Briefs.

[11] Kumar S.D., Esakkirajan S., Bama S., Keerthiveena B. (2020). A microcontroller based machine vision approach for tomato grading and sorting using SVM classifier. Microprocess. Microsyst..

[12] Abrar A., Jalal A., Kim K. Region and decision tree-based segmentations for Multi-objects detection and classification in Outdoor Scenes. Proceedings of the 2019 International Conference on Frontiers of Information Technology (FIT).

[13] Rachana P., Patel S. (2020). A comprehensive study of applying convolutional neural network for computer vision. Int. J. Adv. Sci. Technol..

[14] Bhatt D., Patel C., Talsania H., Patel J., Vaghela R., Pandya S., Ghayvat H. (2021). CNN variants for computer vision: History, architecture, application, challenges and future scope. Electronics.

[15] Esteva A., Chou K., Yeung S., Naik N., Madani A., Mottaghi A., Liu Y., Topol E., Dean J., Socher R. (2021). Deep learning-enabled medical computer vision. NPJ Digit. Med..

[16] Magnus E. (2021). Learning Deep Learning: Theory and Practice of Neural Networks, Computer Vision, Natural Language Processing, and Transformers Using TensorFlow.

[17] O’Mahony N., Campbell S., Carvalho A., Harapanahalli S., Hernandez G.V., Krpalkova L., Riordan D., Walsh J. Deep learning vs. traditional computer vision. Proceedings of the 2019 Computer Vision Conference (CVC).

[18] Ahmed M., Anwar A., Mahmood A.N., Shah Z., Maher M.J. (2015). An investigation of performance analysis of anomaly detection techniques for big data in scada systems. Eai Endorsed Trans. Ind. Netw. Intell. Syst..

[19] Pooja K., Sugandhi R. Anomaly detection for predictive maintenance in industry 4.0—A survey. Proceedings of the 6th International Conference on Energy and City of the Future (EVF’2019).

[20] Susto G.A., Terzi M., Beghi A. (2017). Anomaly detection approaches for semiconductor manufacturing. Procedia Manuf..

[21] Chandola V., Banerjee A., Kumar V. (2009). Anomaly detection: A survey. ACM Comput. Surv..

[22] Bhuyan M.H., Bhattacharyya D.K., Kalita J.K. (2013). Network anomaly detection: Methods, systems and tools. IEEE Commun. Surv. Tutor..

[23] Choi K., Yi J., Park C., Yoon S. (2021). Deep Learning for Anomaly Detection in Time-Series Data: Review, Analysis, and Guidelines. IEEE Access.

[24] Roth K., Pemula L., Zepeda J., Schölkopf B., Brox T., Gehler P. Towards Total Recall in Industrial Anomaly Detection. Proceedings of the IEEE/CVF Conference on Computer Vision and Pattern Recognition.

[25] Ding K., Zhang J., Ding H., Liu Y., Chen F., Li Y. (2020). Fault detection of photovoltaic array based on Grubbs criterion and local outlier factor. Iet Renew. Power Gener..

[26] Gu X., Akoglu L., Rinaldo A. Statistical analysis of nearest neighbor methods for anomaly detection. Proceedings of the 2019 Conference on Neural Information Processing Systems.

[27] Leon-Lopez K.M., Mouret F., Arguello H., Tourneret J.Y. (2021). Anomaly detection and classification in multispectral time series based on hidden Markov models. IEEE Trans. Geosci. Remote. Sens..

[28] Gong D., Liu L., Le V., Saha B., Mansour M.R., Venkatesh S., Hengel A.V.D. Memorizing normality to detect anomaly: Memory-augmented deep autoencoder for unsupervised anomaly detection. Proceedings of the IEEE/CVF International Conference on Computer Vision.

[29] Ahmad Z., Shahid Khan A., Nisar K., Haider I., Hassan R., Haque M.R., Tarmizi S., Rodrigues J.J.P.C. (2021). Anomaly detection using deep neural network for IoT architecture. Appl. Sci..

[30] Omar S., Ngadi A., Jebur H.H. (2013). Machine Learning Techniques for Anomaly Detection: An Overview. Int. J. Comput. Appl..

[31] Zhou Y., Song X., Zhang Y., Liu F., Zhu C., Liu L. (2021). Feature encoding with autoencoders for weakly supervised anomaly detection. IEEE Trans. Neural Netw. Learn. Syst..

[32] Shujian Y., Príncipe J.C. (2019). Understanding Autoencoders with Information Theoretic Concepts. Neural Netw..

[33] Qian J., Song Z., Yao Y., Zhu Z., Zhang X. (2022). A Review on Autoencoder Based Representation Learning for Fault Detection and Diagnosis in Industrial Processes. Chemom. Intell. Lab. Syst..

[34] Charte D., Charte F., del Jesus M.J., Herrera F. (2020). An analysis on the use of autoencoders for representation learning: Fundamentals, learning task case studies, explainability and challenges. Neurocomputing.

[35] Song Y., Hyun S., Cheong Y.G. (2021). Analysis of autoencoders for network intrusion detection. Sensors.

[36] Nguyen D.T., Lou Z., Klar M., Brox T. (2019). Anomaly Detection With Multiple-Hypotheses Predictions. Int. Conf. Mach. Learn..

[37] Liu J., Song K., Feng M., Yan Y., Tu Z., Zhu L. (2021). Semi-supervised Anomaly Detection with Dual Prototypes Autoencoder for Industrial Surface Inspection. Opt. Lasers Eng..

[38] Angelov S., Lazarova M. Convolutional Autoencoders for Image Comparison in Printing Industry Quality Control. Proceedings of the 2022 10th International Scientific Conference on Computer Science (COMSCI).

[39] Kozamernik N., Bračun D. (2020). Visual inspection system for anomaly detection on KTL coatings using variational autoencoders. Procedia CIRP.

[40] Heger J., Desai G., El Abdine M.Z. (2020). Anomaly detection in formed sheet metals using convolutional autoencoders. Procedia CIRP.

[41] Niu T., Li B., Li W., Qiu Y., Niu S. (2021). Positive-sample-based surface defect detection using memory-augmented adversarial autoencoders. IEEE ASME Trans. Mechatron..

[42] Kim J., Ko J., Choi H., Kim H. (2021). Printed circuit board defect detection using deep learning via a skip-connected convolutional autoencoder. Sensors.

[43] Maggipinto M., Beghi A., Susto G.A. (2022). A deep convolutional autoencoder-based approach for anomaly detection with industrial, non-images, 2-dimensional data: A semiconductor manufacturing case study. IEEE Trans. Autom. Sci. Eng..

[44] Ren H., Chai Y., Qu J., Zhang K., Tang Q. An intelligent fault detection method based on sparse auto-encoder for industrial process systems: A case study on tennessee eastman process chemical system. Proceedings of the 2018 10th International Conference on Intelligent Human-Machine Systems and Cybernetics (IHMSC).

[45] Zhu J., Jiang M., Liu Z. (2021). Fault detection and diagnosis in industrial processes with variational autoencoder: A comprehensive study. Sensors.

[46] Davletshina D., Melnychuk V., Tran V., Singla H., Berrendorf M., Faerman E., Fromm M., Schubert M. (2020). Unsupervised Anomaly Detection for X-Ray Images. arXiv.

[47] Mao Y., Xue F.F., Wang R., Zhang J., Zheng W.S., Liu H. (2020). Abnormality detection in chest X-ray images using uncertainty prediction autoencoders. Proceedings of the Medical Image Computing and Computer Assisted Intervention–MICCAI 2020: 23rd International Conference, Proceedings, Part VI 23.

[48] Tekawade A., Liu Z., Kenesei P., Bicer T., De Carlo F., Kettimuthu R., Foster I. 3d autoencoders for feature extraction in X-ray tomography. Proceedings of the 2021 IEEE International Conference on Image Processing (ICIP).

[49] Presenti A., Liang Z., Alves Pereira L.F., Sijbers J., De Beenhouwer J. (2022). Automatic anomaly detection from X-ray images based on autoencoders. Nondestruct. Test. Eval..

[50] Tang W., Vian C.M., Tang Z., Yang B. (2021). Anomaly detection of core failures in die casting X-ray inspection images using a convolutional autoencoder. Mach. Vis. Appl..

[51] Ren J., Ren R., Green M., Huang X. Defect detection from X-ray images using a three-stage deep learning algorithm. Proceedings of the 2019 IEEE Canadian Conference of Electrical and Computer Engineering (CCECE).

[52] Sandler M., Howard A., Zhu M., Zhmoginov A., Chen L.C. MobileNetV2: Inverted Residuals and Linear Bottlenecks. Proceedings of the 2018 IEEE/CVF Conference on Computer Vision and Pattern Recognition.

[53] Deng J., Dong W., Socher R., Li L.J., Li K., Fei-Fei L. ImageNet: A large-scale hierarchical image database. Proceedings of the 2009 IEEE Conference on Computer Vision and Pattern Recognition.

[54] Bergmann P., Batzner K., Fauser M., Sattlegger D., Steger C. (2021). The MVTec Anomaly Detection Dataset: A Comprehensive Real-World Dataset for Unsupervised Anomaly Detection. Int. J. Comput. Vis..

[55] Bergmann P., Fauser M., Sattlegger D., Steger C. MVTec AD—A Comprehensive Real-World Dataset for Unsupervised Anomaly Detection. Proceedings of the IEEE/CVF Conference on Computer Vision and Pattern Recognition (CVPR).

[56] Malesa M., Rajkiewicz P. (2021). Quality Control of PET Bottles Caps with Dedicated Image Calibration and Deep Neural Networks. Sensors.

[57] Yu J., Zheng Y., Wang X., Li W., Wu Y., Zhao R., Wu L. (2021). FastFlow: Unsupervised Anomaly Detection and Localization via 2D Normalizing Flows. arXiv.

[58] Batzner K., Heckler L., König R. (2023). EfficientAD: Accurate Visual Anomaly Detection at Millisecond-Level Latencies. arXiv.

[59] Ahuja N.A., Ndiour I., Kalyanpur T., Tickoo O. (2019). Probabilistic Modeling of Deep Features for Out-of-Distribution and Adversarial Detection. arXiv.

[60] Defard T., Setkov A., Loesch A., Audigier R. (2021). PaDiM: A Patch Distribution Modeling Framework for Anomaly Detection and Localization. International Conference on Pattern Recognition.

[61] Akcay S., Ameln D., Vaidya A., Lakshmanan B., Ahuja N., Genc U. Anomalib: A Deep Learning Library for Anomaly Detection. Proceedings of the 2022 IEEE International Conference on Image Processing (ICIP).

[62] Intel^®^ Distribution of OpenVINO™ Toolkit. https://www.intel.com/content/www/us/en/developer/tools/openvino-toolkit/overview.html.

[63] Kim K., Kim H., Chun J., Kang M., Hong M., Min B. (2021). Real-Time Anomaly Detection in Packaged Food X-Ray Images Using Supervised Learning. Comput. Mater. Contin..

[64] Wang Y., Zhang Y., Zheng L., Yin L., Chen J., Lu J. (2021). Unsupervised Learning with Generative Adversarial Network for Automatic Tire Defect Detection from X-ray Images. Sensors.

